# Cystic Fibrosis-Associated Stenotrophomonas maltophilia Strain-Specific Adaptations and Responses to pH

**DOI:** 10.1128/JB.00478-18

**Published:** 2019-03-13

**Authors:** Tara Gallagher, Joann Phan, Andrew Oliver, Alexander B. Chase, Whitney E. England, Stephen Wandro, Clark Hendrickson, Stefan F. Riedel, Katrine Whiteson

**Affiliations:** a Department of Molecular Biology and Biochemistry, University of California, Irvine, California, USA; b Department of Ecology and Evolutionary Biology, University of California, Irvine, California, USA; Geisel School of Medicine at Dartmouth

**Keywords:** *Stenotrophomonas*, acidic, cystic fibrosis, pH, transcriptome

## Abstract

The airway fluids of cystic fibrosis (CF) patients contain local pH gradients and are more acidic than those of healthy individuals. pH is a critical factor that is often overlooked in studies seeking to recapitulate the infection microenvironment. We sought to determine the impact of pH on the physiology of a ubiqituous yet understudied microbe, Stenotrophomonas maltophilia. Phylogenomics was first used to reconstruct evolutionary relationships between 74 strains of S. maltophilia (59 from CF patients). Neither the core genome (2,158 genes) nor the accessory genome (11,978 genes) distinguish the CF and non-CF isolates; however, strains from similar isolation sources grouped into the same subclades. We grew two human and six CF S. maltophilia isolates from different subclades at a range of pH values and observed impaired growth and altered antibiotic tolerances at pH 5. Transcriptomes revealed increased expression of both antibiotic resistance and DNA repair genes in acidic conditions. Although the gene expression profiles of S. maltophilia in lab cultures and CF sputum were distinct, we found that the same genes associated with low pH were also expressed during infection, and the higher pH cultures were more similar to sputum metatranscriptomes. Our findings suggest that S. maltophilia is not well adapted to acidity and may cope with low pH by expressing stress response genes and colonizing less acidic microenvironments. As a whole, our study underlines the impact of microenvironments on bacterial colonization and adaptation in CF infections.

**IMPORTANCE** Understanding bacterial responses to physiological conditions is an important priority for combating opportunistic infections. The majority of CF patients succumb to inflammation and necrosis in the airways, arising from chronic infection due to ineffective mucociliary clearance. Steep pH gradients characterize the CF airways but are not often incorporated in standard microbiology culture conditions. Stenotrophomonas maltophilia is a prevalent CF opportunistic pathogen also found in many disparate environments, yet this bacterium’s contribution to CF lung damage and its response to changing environmental factors remain largely understudied. Here, we show that pH impacts the physiology and antibiotic susceptibility of S. maltophilia, with implications for the development of relevant *in vitro* models and assessment of antibiotic sensitivity.

## Abstract (precis)

Understanding bacterial responses to physiological conditions is an important priority for combating opportunistic infections. The majority of CF patients succumb to inflammation and necrosis in the airways, arising from chronic infection due to ineffective mucociliary clearance. Steep pH gradients characterize the CF airways but are not often incorporated in standard microbiology culture conditions. Stenotrophomonas maltophilia is a prevalent CF opportunistic pathogen also found in many disparate environments, yet this bacterium’s contribution to CF lung damage and its response to changing environmental factors remain largely understudied. Here, we show that pH impacts the physiology and antibiotic susceptibility of S. maltophilia, with implications for the development of relevant *in vitro* models and assessment of antibiotic sensitivity.

## INTRODUCTION

The ability of a microbe to successfully colonize and persist in a new environment depends on its tolerance of various conditions. pH is a central environmental factor that imposes selective pressure on bacterial phyla and species (1–4), drives shifts in microbial metabolism (3, 5), and affects microbial interactions (3, 6). pH response is considered a deeply conserved trait (4), where different bacteria have specific pH ranges at which they reach dense growth. For example, Gram-negative opportunistic pathogens, including Pseudomonas aeruginosa, grow optimally at neutral pH, yet must survive at growth-limiting pH in the environment and infections (7, 8).

One example of infection-relevant pH shifts concerns the airway secretions of cystic fibrosis (CF) patients, which are characterized by steep pH gradients that can suppress bacterial growth. The pH of CF sputum ranges from 2.9 to 6.5 (9), although transient microenvironments of alkaline pH likely exist, arising from bacterial metabolism of amino acids (10, 11). pH is decreased by the CFTR (cystic fibrosis transmembrane conductance regulator) bicarbonate channel defect (12–14), and the pH can be further reduced during periods of decline in CF lung function, known as pulmonary exacerbations (15–17), potentially due to both host and microbial production of acidic molecules, such as lactic acid (16).

Chronic bacterial colonization in the airways can result in up to a 95% mortality rate in CF patients (18); antibiotics are rarely capable of eradicating established bacterial infections. To better inform treatment, it is imperative to understand the mechanisms opportunistic pathogens use to persist in the airways. The effect of pH on CF bacteria is vastly understudied despite its importance as a major environmental factor in microbial communities. One such CF microbe is Stenotrophomonas maltophilia, which is estimated to infect 10% to 18% of patients (19, 20) and is intrinsically resistant to multiple antibiotic classes. S. maltophilia is unable to use nitrate as an alternative electron acceptor (21), a trait that likely impacts its growth and colonization location in the airways (22). One recent retrospective study found that baseline chronic S. maltophilia infection is associated with a 3-fold increased risk of mortality or lung transplant in CF patients (23). Two recent studies found that CF-associated S. maltophilia has a wide pangenome (24), and human-associated S. maltophilia forms core genome clades that are distinct from environmental strains (25). In both studies, there was little correlation between genetic potential and observed phenotypes in S. maltophilia (24, 25), including antibiotic susceptibility, which further emphasizes a need to improve the link between genetic information and bacterial physiology in the CF airways. Our knowledge of how opportunistic pathogens behave in CF sputum is limited. To date, only a few studies have looked at changes in CF bacterial gene expression *in vivo* (26–28). The lack of overlap between experimental conditions and CF sputum is an important factor in the observed differences in bacterial physiology *in vitro* versus *in vivo* (27).

Here, we used a combination of core genome phylogenetics, pangenome analyses, and CF sputum metatranscriptomics, along with transcriptomics, growth curves, and antibiotic assays in a range of pH. We hypothesized that S. maltophilia responds to acidic conditions in the CF airways by acquiring and expressing stress response genes. Our phylogenomics analyses did not support that S. maltophilia CF strains acquire a specific universal adaptation to acidic pH. Rather, our combined phylogenomic and transcriptomic analyses indicate S. maltophilia utilizes both conserved and strain-specific stress responses in lower pH. Furthermore, higher pH cultures had more similar transcriptomes to those of sputum than those of acidic pH cultures, suggesting that S. maltophilia may avoid or have limited growth in the lower-pH microenvironments in CF sputum. Our study highlights a need for better *in vitro* systems, as well as showing the impact of pH on the localization and adaptation of CF bacteria.

## RESULTS

### S. maltophilia core genome analyses.

**FIG 1:**
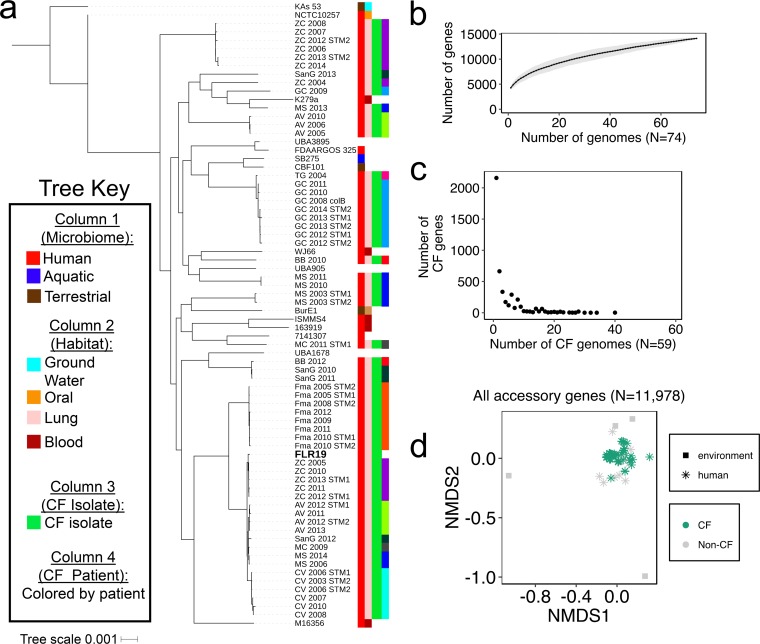
S. maltophilia core and pangenome analyses. (a) Phylogenetic tree for 74 S. maltophilia strains constructed using 2,158 genes conserved across all strains. Information about the strains’ isolation source is represented by the colored blocks in the four columns next to the tree. The last column highlights clonal strains isolated from the sputum of ten CF patients from Esposito et al.’s 2017 study (24), where each patient is designated by a different color and by the first portion of the genome name. (b) S. maltophilia pangenome accumulation plot (14,136 gene clusters, *n* = 100 permutations). (c) The distribution of genes found only in CF genomes (4,457 genes). No CF-specific genes were shared in all 59 CF isolates. In accordance with this, the accessory gene content (*n* = 11,978 genes) did not strongly separate strains into CF and non-CF groups. (d) NMDS of Jaccard dissimilarity matrix of accessory genome content (*n* = 11,978 genes, stress = 0.16), where the color indicates if strains were isolated from CF patients (*n* = 59) or were non-CF strains (*n* = 15). The shape indicates if a strain came from a human (*n* = 70) or was environmental (*n* = 4) sample. An ANOSIM of the Jaccard dissimilarity matrix suggested that the accessory genome is not a strong indicator of whether a strain is CF or non-CF (*R* = 0.55, *P* < 0.05). A nested PERMANOVA of the Jaccard dissimilarity matrix indicated that the patient from whom a strain originates explains more of the variation (*R*^2^ = 37%) in accessory genome content than whether the strain originated from a CF patient (*R*^2^ = 5.5%) (*P* < 0.001).

To determine the relationships among CF strains, we conducted a phylogenomics analysis using all known S. maltophilia genomes (153 genomes [see Data Set S1 in the supplemental material]). This analysis identified 74 closely related strains representing a diverse genome set, including environmental and host-associated strains. For all downstream genomics analyses, we grouped genes with a minimum 95% amino acid identity (AAI) into a cluster (here designated “gene”). By concentrating on the core genome (*n* = 2,158), genes shared among all analyzed genomes, we found that most of the CF isolates formed highly similar phylogenetic subclusters (Fig. 1a). In general, clonal strains isolated from the same CF patient (24) were closely related and found in the same subclade. In contrast, the environmental strains had larger differences in their core genomes, evidenced by deeper branch lengths in the tree.

### S. maltophilia cystic fibrosis accessory genes.

In order to identify genes unique to S. maltophilia FLR19 and/or other CF isolates, we analyzed the pangenome, genes that make up the core and accessory genomes (Data Set S2). S. maltophilia has an open pangenome consisting of 14,136 genes (Fig. 1b). The average number of genes in each S. maltophilia genome was 4,285 (minimum = 3,908 genes in strain UBA905, maximum = 4,733 genes in strain GC 2009) (Fig. S1). The CF accessory genome (genes found only in the 59 CF strains) consisted of 4,457 genes, although no gene was found across all 59 CF genomes. Only five of the CF-specific genes, which had no known function, were shared in at least half of the CF isolates (Data Set S2; Fig. 1c). Our analyses suggest that the genes comprising the CF accessory genome are strain specific, with nearly half (*n* = 2,157 CF-specific accessory genes) being unique to one strain (Fig. 1c). Accessory genome content was better explained by patient identifier (*R*^2^ = 37%) than CF status (*R*^2^ = 5.5%) (nested permutational multivariate analysis of variance [PERMANOVA]; *P* < 0.001). In accordance with this, the accessory gene content (*n* = 11,978 genes) moderately separated strains into CF and non-CF groups (analysis of similarity [ANOSIM]; *R* = 0.55, *P* < 0.05) (Fig. 1d).

We next examined whether the overall functional potential of CF strains differed from that of non-CF strains. In particular, we hypothesized that CF strains of S. maltophilia would be enriched for stress response genes to cope with acidic pH in the CF airways. We grouped total (core and accessory) gene content into 26 functional categories based on the combined Rapid Annotation using Subsystem Technology (RAST) SEED annotations of a non-CF type strain (K279a) and a CF isolate (FLR19) (29) (Data Set S3). CF strains did not contain significantly more genes in the “stress response” category but did have greater proportions of genes in the “virulence” and “cofactors” categories (Fig. S1B; Data Set S4) (two-sample *t* test; *P* < 0.05). The annotated functions of genes in the virulence category included metal and antibiotic resistance (Data Set S3).

### S. maltophilia growth in acidic, neutral, and basic pH with antibiotics.

**FIG 2:**
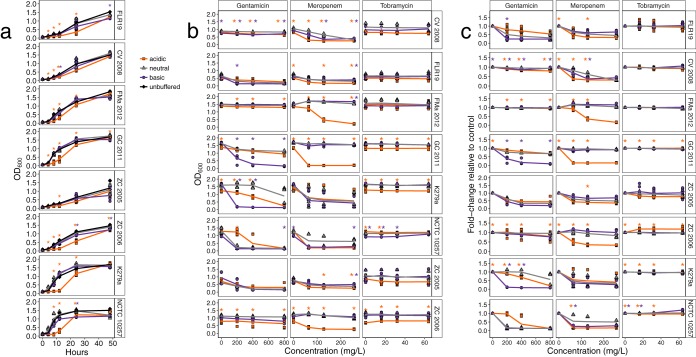
Growth curves of eight S. maltophilia strains, consisting of six CF isolates (FLR19, CV 2008, FMa 2012, GC 2011, ZC 2005, and ZC 2006) and two non-CF human strains (K279a and NCTC 10257). Orange asterisks indicate a significant pairwise comparison in acidic to neutral pH, while purple asterisks indicate a significant comparison in basic to neutral pH. (a) The strains were cultured in acidic (initial pH 5), neutral (initial pH 7), and basic (initial pH 9) buffered media (*n* = 6 to 9 replicates; the line represents averages of replicates). As a comparison, the strains were also cultured in unbuffered media (initial pH, 7.8). Asterisks indicate significant results with an ANOVA and *post hoc* pairwise comparisons, (*P* < 0.05). (b) Growth of S. maltophilia strains in pH-buffered media with different concentrations of gentamicin, tobramycin, and meropenem after 24 h of incubation. The line represents averages from replicates (*n* = 6 to9 replicates). Asterisks represent *P* < 0.05 from ANOVA with *post hoc* comparisons and Bonferroni correction. (c) Fold change in the growth of each strain with antibiotics compared to growth without antibiotics. Asterisks represent *P* < 0.05 from two-sample *t* tests with Bonferroni corrections of the fold change values for acidic and basic pH compared to neutral pH.

Because the pH of CF airways is acidic and further reduced during pulmonary exacerbations, we wanted to determine how S. maltophilia responds to changes in pH. We grew six CF isolates (from San Diego, CA, and from Italy) (24) and two non-CF strains in phosphate-buffered pH 5, 7, and 9 Todd-Hewitt broth. All eight strains had impaired growth in acidic pH relative to that in neutral pH (analysis of variance [ANOVA] with *post hoc* pairwise comparisons; *P* < 0.05) (Fig. 2a). Similar results were obtained in nonbuffered acidic media spiked with citric, lactic, or sulfuric acid (Fig. S2A). Cells recovered from a medium at a specific pH were not more tolerant to growth at that pH, suggesting that their growth was due to a physiological response rather than to mutational adaptation to acidic tolerance (Fig. S2B). In addition, strain FLR19 increased its local pH in the pH 5 buffered Todd-Hewitt broth over a 24-h growth period (Fig. S2C). The growth of strain FLR19 growth was also impaired in acidic artificial sputum media (ASM) (Fig. S2D).

Beyond observing pH-driven changes in growth, we assayed tolerance to antibiotics prescribed to CF patients (gentamicin, tobramycin, and meropenem) across the pH gradient (Fig. 2b). The bacterial susceptibility to meropenem and gentamicin in different pH varied at the strain level (ANOVA with *post hoc* comparisons and Bonferroni correction; *P* < 0.05, *n* = 6 to 9). Five of the CF strains (FLR19, FMa 2012, CV 2008, GC 2011, and ZC 2006) were more susceptible to meropenem in acidic pH than in neutral pH (Fig. 2c) (*t* test with Bonferroni correction; *P* < 0.05, *n* = 6 to 9). The reverse pH effect was observed with gentamicin for four strains (FLR19, GC 2011, ZC 2005, and K279a), which were more susceptible in basic pH than in to neutral pH. Strain NCTC 10257 had significantly increased tolerance of gentamicin, and strain FLR19 trended toward increased tolerance of gentamicin in acidic conditions. These findings align with those of previous antibiotic assays, which showed that β-lactams (meropenem) have increased activity at lower pH, while aminoglycosides (gentamicin) show decreased activity (30).

### S. maltophilia FLR19 metabolome under acidic, neutral, and basic pH conditions.

**FIG 3:**
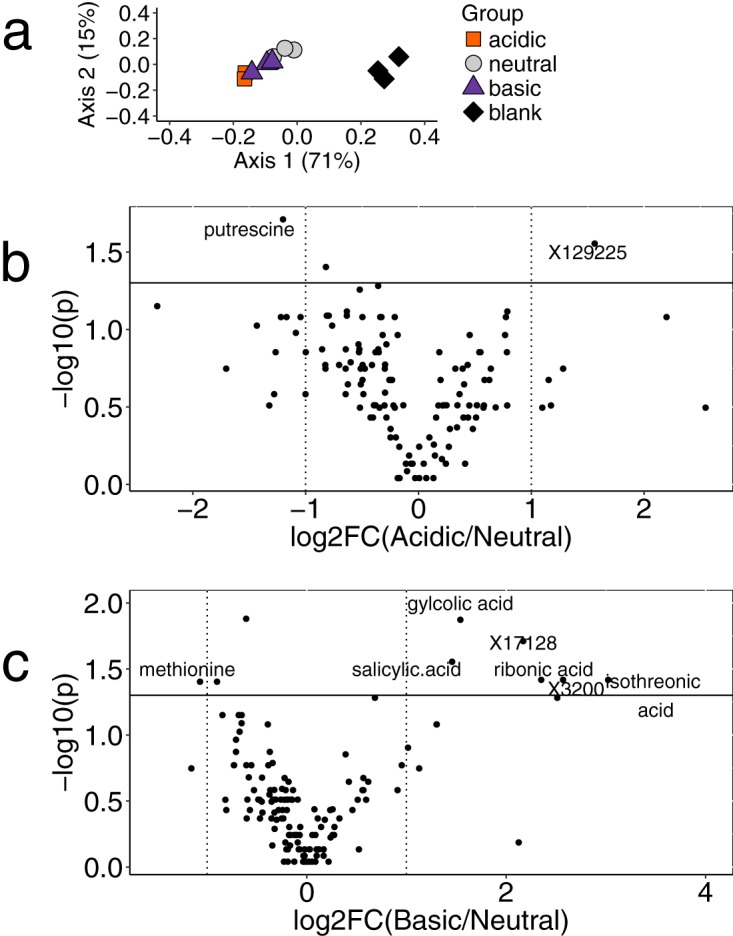
Metabolomics of strain FLR19 under different pH conditions. (a) Principal coordinate ordination analysis (PCoA) of Bray-Curtis distances of the metabolite abundances for the uninoculated medium blank and S. maltophilia FLR19 grown in acidic pH, neutral pH, and basic pH (*n* = 3 replicates). (b and c) Volcano plots of the log_2_ fold change (log_2_FC) difference in metabolite abundance for the acidic metabolome (b) and basic metabolome (c) relative to the neutral metabolome. Negative log_10_(p) is the *P* value from the *post hoc* Dunn analysis, where a *P* value of < 0.05 and a log_2_FC value of >1 or <−1 were considered to be significant. Metabolites with an “X” prefix were nonannotated.

We next wanted to determine how S. maltophilia responds metabolically and transcriptionally to changes in pH. We chose FLR19 for the metabolomics and transcriptomics because it is a CF strain not yet characterized but is still closely related evolutionarily to 27 other CF strains from our core genome phylogenetics (Fig. 1a). Metabolites that were produced or consumed in different conditions were identified using untargeted metabolomics. The metabolomes had little separation based on pH but had distinct metabolic profiles from uninoculated media (Fig. 3a). S. maltophilia FLR19 produced 226 metabolites in at least one of the experimental conditions; 40 metabolites were produced in all three conditions (Data Set S4).

S. maltophilia FLR19 produced 28 metabolites in acidic medium only. These included hydroxyglutaric acid, a by-product of glutamate catabolism (31), and hydroquinone and acetophenone, weak acids with high pK_a_. Notably, metabolites involved in polyamine synthesis, *N*-acetylglutamate, putrescine, and spermidine, were consumed by FLR19 in acidic conditions (Data Set S4). The acidic metabolome had significantly less putrescine and more of a nonannotated metabolite (X129225) compared to the neutral metabolome (Kruskal-Wallis ANOVA with *post hoc* Dunn comparisons; *n* = 3, *P* < 0.05, log_2_ fold change [log_2_FC] > 1 or < −1) (Fig. 3b; Fig. S4). The production of polyamines via decarboxylation of amino acids is a well-documented acidic stress response in bacteria (32). The FLR19 strain’s consumption of *N*-acetylglutamate, spermidine, and putrescine in acidic pH could be indicative of higher turnover of those intermediates in this polyamine pathway.

Metabolites produced by S. maltophilia FLR19 in alkaline pH included weak acids, such as ribonic acid, salicylic acid, urea, glycolic acid, glyceric acid, and isothreonic acid. The basic metabolome had significantly less methionine and more organic acids (Kruskal-Wallis ANOVA with *post hoc* Dunn comparisons; *P* < 0.05, *n* = 3, log_2_FC > 1 or < −1) (Fig. 3c; Fig. S4).

### S. maltophilia FLR19 transcriptome in acidic, neutral, and basic pH.

**FIG 4:**
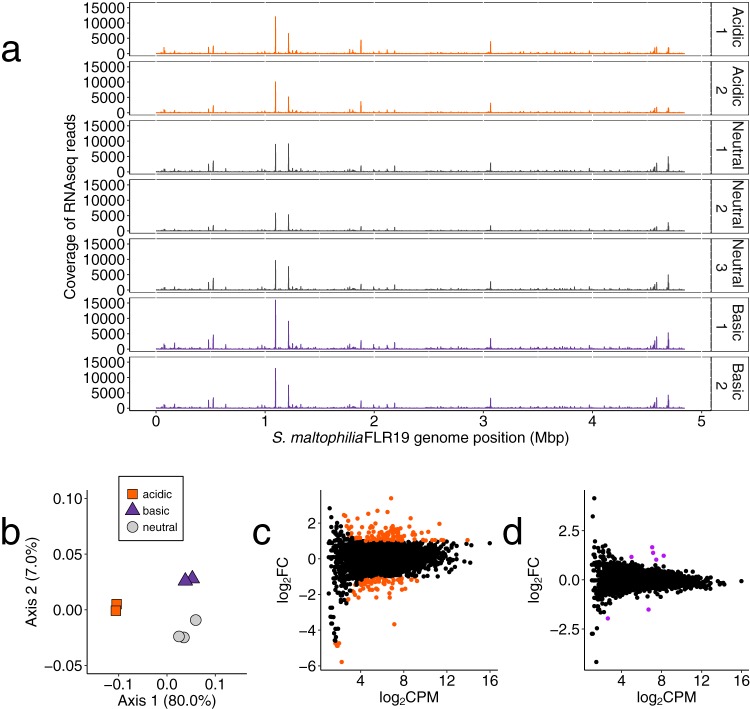
Differential gene expression analyses of FLR19 grown under a range of pH conditions. (a) Coverage of transcriptome reads (counts per million) across the S. maltophilia FLR19 genome. (b) Principal-coordinate analysis (PCoA) of the Bray-Curtis distance matrix of the *in vitro* S. maltophilia transcriptomes (*n* = 2 for acidic and basic transcriptomes and *n* = 3 for neutral transcriptomes). (c and d) Smear plots of genes in the acidic transcriptome compared to the neutral transcriptome (c) and basic transcriptome compared to the neutral transcriptome (d). Each dot represents the log_2_ counts per million (log_2_CPM) average from both the replicates (*x* axis) and the average log_2_FC of the acidic or basic CPM divided by neutral CPM (*y* axis). Colored dots indicate a log_2_FC of >1 or <−1 and an FDR of <0.05.

In order to determine how our CF isolate responds transcriptionally to changes in pH, we sequenced ribosome-depleted RNA from FLR19 cultures grown in acidic (*n* = 2), neutral (*n* = 3), or basic pH (*n* = 2). The numbers of quality-filtered reads that aligned to the FLR19 genome were 2.2 to 5.1 million, with mean genome-wide coverages ranging from 27× to 67× (Fig. 4a; Data Set S6). The transcriptomes separated based on pH, and the acidic transcriptomes are more distinct from the neutral than the basic transcriptomes (axis 1) (Fig. 4b). The acidic transcriptome had 86 upregulated genes and 84 downregulated genes (negative binomial test; false-discovery rate [FDR] < 0.05, log_2_FC > 1 or < −1) (Fig. 4c; Data Set S6). The basic transcriptome only had five upregulated genes and two downregulated genes (Fig. 4d; Data Set S6). These results suggest that S. maltophilia FLR19 is better suited to grow in basic pH than in acidic pH, which is consistent with the growth curve data.

### Comparison of S. maltophilia gene expression profiles *in vitro* and in CF sputum.

In order to identify genes actively transcribed by S. maltophilia in CF airways, we mapped metatranscriptome reads from sputum (taken from 7 CF patients infected with P. aeruginosa [27]) to the S. maltophilia pangenome (all coding sequences from 74 strains). Before aligning the metatranscriptome reads to the S. maltophilia pangenome, the reads were mapped to a custom-made CF database consisting of 1,812 non-Stenotrophomonas CF bacterial genomes from the Pathosystems Resource Integration Center (PATRIC) (33). While this approach reduced the number of false-positive hits to the S. maltophilia pangenome from other bacterial RNAs, it also omitted multispecies genes. Two of the sputum samples (E and F) had reads that aligned to S. maltophilia genes (3,403 and 28,992 reads, respectively) (Data Set S5).

**FIG 5:**
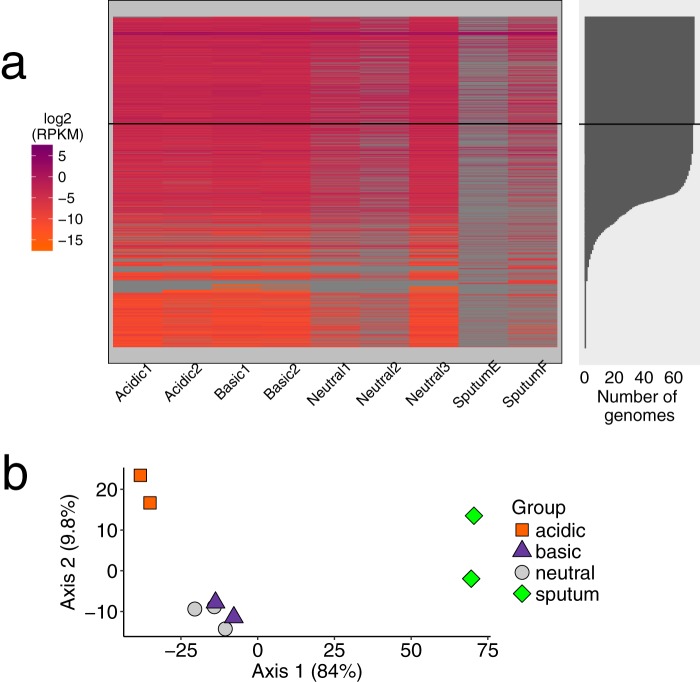
Gene expression *in vitro* and in CF sputum. (a) Heat map of the log_2_ reads per kilobase of transcript per million mapped reads (RPKM) values of the transcriptome and metatranscriptome reads aligned to the S. maltophilia pangenome. Each row is one gene (95% AAI clusters from pangenome analyses). The number of S. maltophilia genomes which have that gene is indicated on the right. Any gene above the black horizontal line in both plots was part of the core genome (found in all 74 strains). (b) PCoA plot of the Euclidean distance matrix of RPKM values of 918 genes expressed in all samples.

To compare transcription of S. maltophilia FLR19 grown *in vitro* to that of S. maltophilia in sputum, we first processed the *in vitro* RNA sequencing reads through the same pipeline as the metatranscriptome reads. Fewer genes were detected in the CF metatranscriptomes than in the *in vitro* transcriptomes (Fig. 5a; Data Set S6). Since the CF airways are primarily acidic (9), we hypothesized *a priori* that the acidic transcriptomes would be similar to the sputum metatranscriptomes. However, the gene expression profiles of the acidic transcriptomes were least similar to the CF sputum metatranscriptomes (Fig. 5b). The source of the RNA (*in vitro* versus *in vivo*) explained more variance in the gene expression profiles (*R*^2^ = 0.79) than did the experimental pH (*R*^2^ = 0.14) (nested PERMANOVA; *P* < 0.05).

**FIG 6:**
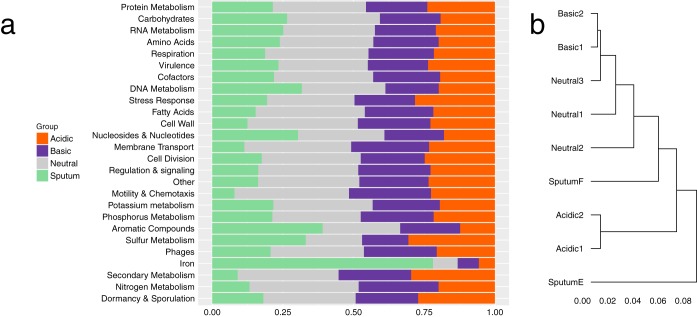
Functional activities of FLR19 under a range of pH conditions and of S. maltophilia in CF sputum. (a) Relative proportion of RNA sequencing reads that aligned to 26 functional categories from the sputum transcriptomes (*n* = 2) and the *in vitro* acidic (*n* = 2), neutral (*n* = 3), and basic pH transcriptomes (*n* = 2). (b) Hierarchical clustering by sample of the Euclidean distance matrix of proportion of reads that aligned to a functional category.

Determining the proportion of metatranscriptome and transcriptome reads that aligned to functional categories (29) showed that “protein metabolism” was the most abundant category across all samples (Fig. 6a). Overall, there were few changes in the rankings of the categories except for minor differences (Data Set S3; Fig. 6a). Notably, the sputum metatranscriptomes had a higher proportion of reads that aligned to genes involved in iron acquisition (sputum E = 1.2%, sputum F = 0.5%) than did the *in vitro* transcriptomes (0.02% to 0.08%) (Data Set S3). Hierarchical clustering of the enriched categories indicated that the sputum samples were functionally more similar to the alkaline and neutral cultures than to the acidic cultures (Fig. 6b).

### Identification of pH response genes expressed *in vitro* and in CF sputum.

**FIG 7:**
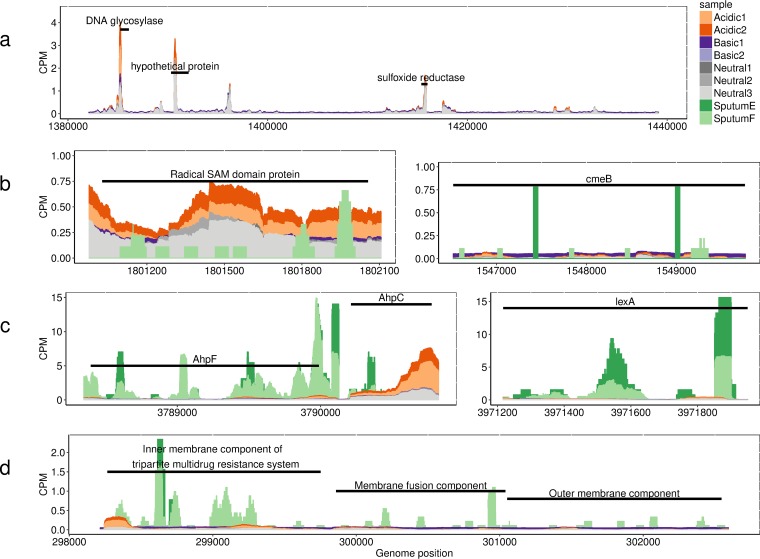
Examples of genes expressed in acidic pH and CF sputum. The black bars in the plots depict the lengths of genes. CPM, counts per million. (a) Coverage of S. maltophilia FLR19 transcriptome reads across a 58,000-bp region which contained some genes unique to our strain. The labels indicate genes with higher expression in acidic pH. The DNA glycosylase was significantly upregulated in acidic pH compared to neutral pH. (b) FLR19 transcriptome reads and sputum metatranscriptome reads across a radical SAM domain protein (left panel) closely related to a DNA lyase and found in six S. maltophilia CF isolates and across the RND efflux pump CmeB (right panel). (c) FLR19 transcriptome reads and sputum metatranscriptome reads aligned to the genes for alkyl hydroperoxide reductases (AhpF and AhpC) and the SOS response repressor LexA, genes that were conserved across all 74 S. maltophilia strains. (d) FLR19 transcriptomes and sputum metatranscriptomes across three components of the tripartite multidrug efflux system.

By combining our pangenome analyses and transcriptomics, we identified a DNA glycosylase that was unique to FLR19 and upregulated in acidic pH (Fig. 7a), suggesting that the expression of this DNA glycosylase may be a strain-specific response to low pH. This gene was in a region containing additional genes that were also expressed at higher levels in acidic pH than in neutral pH, but not at statistically significant levels. This included a hypothetical protein and sulfoxide reductase (Fig. 7a).

Two additional accessory genes were upregulated by FLR19 in acidic conditions (relative to neutral pH conditions). The first gene, which was also expressed in sputum F (Fig. 7b, left), encodes a radical *S*-adenosyl-l-methoniine (SAM) domain protein. A SmartBLAST search of the amino acid sequence indicated that this protein is closely related to bacterial photolyases involved in DNA repair. The second gene, also expressed in CF sputum samples, was a multidrug efflux pump gene, *cmeB* (Fig. 7b, right). The gene for the RND CmeB efflux pump was also found in S. maltophilia environmental strains.

Twenty core genes were upregulated by strain FLR19 at low pH and expressed in sputum, including the coding sequences for alkyl hydroperoxide reductases (AhpF and AhpC), the SOS response regulator LexA, and the tripartite multidrug resistance system (Fig. 7c and d; Data Set S7). In a similar study, the same stress response genes were also expressed by P. aeruginosa at high levels in CF sputum (27). None of the seven differentially expressed genes from when FLR19 was grown at basic pH were found to be expressed by S. maltophilia in sputum.

## DISCUSSION

In order to improve treatment strategies, we need to better understand how opportunistic pathogens are capable of living in the dynamic, stressful environments found in the CF airways. The pH of CF sputum ranges from 2.9 to 6.5 (9), and further drops during periods of pulmonary exacerbation (15). Interestingly, many of the common Gram-negative opportunistic pathogens that persist in CF infections have impaired growth at lower pH (7, 8), which motivated us to determine how S. maltophilia copes in nonoptimal, acidic pH. We hypothesized that because the pH of the CF airways is largely acidic, S. maltophilia copes by acquiring and prioritizing expression of stress response genes.

### CF strains of S. maltophilia adapt to patient-specific factors and are not better adapted to low pH.

We first analyzed all publicly available S. maltophilia genomes, along with a clinically relevant isolate unique to this study. For the phylogenomics analysis, we used a 95% AAI cutoff, which allowed us to look at finer-scale relationships between closely related strains but which also potentially overestimated the number of genes in the pangenome. Most of the S. maltophilia CF strains were part of four tight subclades on the phylogenetic tree. Steinmann et al. found that core single-nucleotide polymorphism (SNP) phylogenomics separated Stenotrophomonas spp. into human-associated and environmental clades (25). However, we cannot confirm this trend due to the limited number of environmental genomes that made it through our initial genome-filtering step. The open pangenome of S. maltophilia suggests that this species has diverse capabilities. This is in accordance with results of another study, which looked at the longitudinal phenotypic and genotypic heterogeneity of 91 S. maltophilia isolates from 10 CF patients, where the CF strains had a narrow core genome that made up a fraction of a large pangenome (1,911 core genes out of a total 16,486 genes) (24).

We hypothesized *a priori* one mechanism that S. maltophilia uses to survive low pH is acquiring stress response genes in the CF airways. However, we were unable to identify a CF-specific signal of adaptation to low pH. Our phylogenomics analyses suggest that S. maltophilia adapts to specific niches within a patient’s airways. As such, a single strain cannot be considered representative of the entire CF population for a species. The lack of clonal epidemiology in isolates among CF patients is also seen in P. aeruginosa (34, 35). CF opportunistic pathogens are thought to be acquired from the environment and to colonize the airways of a patient throughout the patient’s life, driving patient-specific adaptation (34–37). Only when analyzing the accessory genome at the functional level (achieved by binning genes into cellular categories) were we able to find that nonessential genes, including those canonically defined as virulence genes, were enriched in CF strains. Furthermore, there was no significant increase in stress response genes in CF isolates. While we did not find a universal genomic adaptation to acidic pH stress in CF strains, the data from the *in vitro* growth experiments suggest that acidic conditions are stressful for S. maltophilia. All eight strains, including six CF isolates and two human strains, had impaired growth at lower pH.

### S. maltophilia can cope with low pH by expressing both strain-specific and conserved stress response genes.

Based on our transcriptomics and metatranscriptomics analyses, S. maltophilia copes with acidic pH by utilizing both universal responses (expression of core genes) and strain-specific responses. One possible mechanism is by increasing transcription of DNA repair genes, as we observed from our transcriptomics analysis. Strain FLR19 contained both strain-specific and core repair genes. The expression of DNA repair genes has been previously identified in sputum metatranscriptomes and likely reflects bacterial response to stressful conditions in the cystic fibrosis airways that can damage DNA, including the presence of reactive oxygen species and antibiotics (5). Consistent with this was FLR19’s increased transcription of alkyl hydroperoxide reductase genes and the stress response gene *lexA*. In a similar study, these stress response genes were upregulated by P. aeruginosa in sputum and conferred resistance to antibiotics that included gentamicin (27). Taken together, the findings of Cornforth et al. and our own findings suggest that CF strains survive in CF-relevant conditions, including acidic pH, with both conserved and adaptive traits.

### Transcriptomics suggest that S. maltophilia may avoid lower pH.

We used a conservative approach to align the sputum metatranscriptomes to the pangenome to ensure that we only included S. maltophilia RNA in our study, which may have resulted in the loss of multispecies signals. We also recognize that the differences in sequencing depth between the *in vitro* transcriptomes and sputum metatranscriptomes bias the identification and quantification of gene expression, and we sought to reduce this bias by calculating the proportion of reads that mapped to functional annotation categories. Overall, the gene expression profiles of the *in vitro* FLR19 cultures were distinct from the S. maltophilia transcriptomes in CF sputum.

In contrast to our gene level analysis, the functional profiles of S. maltophilia in sputum and under the different pH conditions were similar. A couple of categories were more enriched in the sputum transcriptomes than in the *in vitro* transcriptomes, including iron acquistion. While ferritin is abundant in CF sputum, free iron may be scarce, and bacteria utilize scavengers to obtain iron (38, 39). Although expression of iron uptake genes appears to be a priority for S. maltophilia in sputum, we did not see significant enrichment of iron genes from our pangenome analyses in the CF strains, suggesting that S. maltophilia utilizes core genes to acquire iron. The acidic transcriptomes had expressed more stress response genes, indicating that the experimental acidic conditions were stressful for FLR19. Perhaps the pH 5 buffered medium was more stressful for strain FLR19 than sputum, in which S. maltophilia may be capable of increasing local pH and colonizing the higher-pH regions (reported to be as high as 6.5 in pediatric sputum) (9).

We originally hypothesized that the acidic transcriptome would be more similar than the neutral and alkaline transcriptomes to that of S. maltophilia in sputum, because the pH of CF sputum is acidic (with gradients of 2.9 to 6.5) (9). However, clustering the samples by their functional categories indicated that the basic and neutral transcriptomes were more closely related than the acidic transcriptome to that of sputum. The lack of similarities in functional activity between the acidic and sputum transcriptomes, in addition to dissimilar gene expression profile in sputum compared to all *in vitro* conditions, emphasizes our need to better understand how the local environment impacts S. maltophilia colonization. Perhaps the neutral and alkaline transcriptomes were more similar to CF metatranscriptomes because S. maltophilia avoids lower pH microenvironments in CF sputum. In accordance with this, we did not find a strong signal of CF-specific or low-pH adaptations from our phylogenomics analysis. As S. maltophilia is unable to undergo nitrate respiration (21), oxygen is another factor that can determine the success and location of S. maltophilia colonization in the CF airways. In anaerobic environments, bacterial and host cells undergo fermentation, further decreasing the local pH. Cowley et al. reported drops in oxygen and pH with sputum plug depth (9). A recent study finding that Gram-negative opportunistic pathogens prefer regions with higher pH and oxygen levels in sputum mesocosm supports our idea that S. maltophilia colonizes microenviornments that are less acidic and more aerobic (8).

Another possibility for the differences in gene expression in sputum compared to that *in vitro* is the lack of overlap in bacterial behavioral studies *in vitro* versus in sputum. Cornforth et al. highlighted this need, finding a discordance in the expression of gene classes when P. aeruginosa is grown *in vitro* in comparison to that of P. aeruginosa found in human samples, including the same CF sputum samples used in this study (27).

### pH affects S. maltophilia antibiotic tolerances.

One of very few studies that have looked at the effect of pH on cystic fibrosis strains showed that reductions in the pH of airway secretions inhibited its antibacterial function (12). We saw changes in expression of antibiotic resistance genes by strain FLR19 and in antibiotic susceptibility depending on the strain, antibiotic used, and pH. Both CF and non-CF strains were more sensitive to meropenem in acidic conditions. One non-CF strain (NCTC 10257) and on CF strain (FLR19) had higher tolerance of gentamicin at low pH. We also saw increased expression of antibiotic resistance genes (both core and accessory) when FLR19 was grown at low pH. The same antibiotic resistance genes were expressed by S. maltophilia in CF sputum. As CF patients take antibiotics throughout their lives, it is not surprising that a CF isolate expresses antibiotic resistance genes in sputum. While it is known that pH affects antibacterial activity (30), our findings have clinical implications for the treatment of CF infections, especially during pulmonary exacerbations, when the pH of CF airways becomes more acidified (15).

### Conclusion.

**FIG 8:**
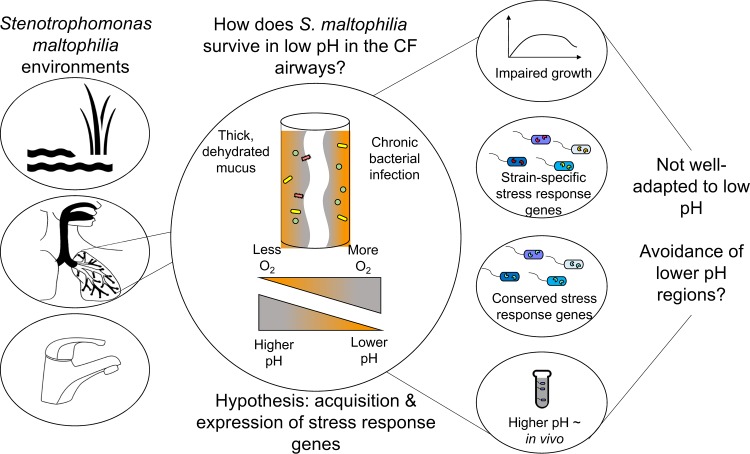
Conceptual overview of study. S. maltophilia is a ubiquitous organism, found in aquatic and soil environments and in cystic fibrosis (CF) infections. Because CF sputum is characterized by gradients of pH (reported to range from 2.9 to 6.5) and oxygen (9), we wanted to determine how S. maltophilia survives under low-pH conditions. Growth assays indicate that low pH is stressful for both CF and non-CF strains. While S. maltophilia can cope with low pH by expressing conserved and adapted stress response genes, our *in vitro* and *in vivo* transcriptomics analyses suggest that S. maltophilia may survive in the airways by avoiding lower-pH microenvironments. Taken together, the results of our study highlight that pH can drive S. maltophilia physiology by inducing stress response mechanisms and controlling the physical colonization of S. maltophilia.

Our results suggest that S. maltophilia is not well-adapted to low pH and uses stress response mechanisms and location to cope with pH gradients characteristic of the CF airways (Fig. 8). Tools that spatially resolve bacteria *in vivo* will be indispensable in understanding where and how bacteria adapt to clinical infections (40).

## MATERIALS AND METHODS

### S. maltophilia FLR19 genome.

S. maltophilia FLR19 was isolated from the sputum of an adult CF patient. The genome was sequenced on an Illumina MiSeq instrument and assembled using A5 (06-04-2016 version) with default parameters. Short scaffolds (<5,000 bp) containing repeated nucleic acid sequences were removed from the genome for downstream analyses.

### Phylogenetic analyses.

To examine the phylogenetic relatedness of our isolate, we constructed an initial S. maltophilia phylogeny using 21 conserved single-copy marker genes. Specifically, we downloaded 153 strains designated S. maltophilia from the PATRIC genome database that contained corresponding metadata (see Data Set S1 in the supplemental material) (33). Next, we screened each downloaded genome for the presence of 21 marker genes with HMMER (41) and built the initial phylogenetic tree with FastTree2 (the Interactive Tree Of Life [iTOL] input for which can be downloaded from the following GitHub repository: https://github.com/tgallagh/Stenotrophomonas) (42). Based on the robustness of the resulting phylogeny, we calculated whole-genome pairwise comparisons (both nucleotide and amino acid identity [AAI]) across all genomes and selected a subset of strains (*n* = 74) that were closely related to our strain (>97% AAI) for downstream analysis. Coding regions from the resulting 74 genomes were translated using Prodigal (43) with predicted functional annotation assigned by Prokka (44). We assigned orthologous protein groups (orthologs) based on a reciprocal protein BLAST search using Roary and clustered orthologs at 95% AAI (45). Single-copy orthologs conserved across all isolates (*n* = 2,158) were used to build a core genome phylogeny. Specifically, each core ortholog was independently aligned using Clustal Omega v1.2.0 (46) and used to create a concatenated core genome alignment (714,427 amino acids). Finally, we constructed a maximum likelihood phylogenetic tree using RAxML v8.0.0 (47) under the “PROTGAMMWAG” model for 100 replicates. We mapped all isolation source data retrieved from the PATRIC metadata onto the tree using iTOL (48). To identify genes unique to S. maltophilia FLR19 and other CF isolates, we compared total gene profiles across the 74 closely related genomes. Geneparser was used to determine the presence or absence of genes within the pangenome for each genome. These pangenome data were visualized using a script developed for the Roary pipeline (45). A cumulative gene plot was made using the “specaccum” function from the R package “vegan.” To determine if accessory gene content is a strong predictor of a strain’s isolation environment, a Jaccard dissimilarity matrix of the accessory genome presence-absence matrix was calculated with the “vegdist” function from the R package “vegan.” The dissimilarity matrix was visualized with a nonmetric multidimensional scaling (NMDS) plot constructed with the “metaMDS” function from “vegan.” An ANOSIM and nested PERMANOVA of the Jaccard dissimilatory matrix were conducted using the “anosim” and “adonis” functions from “vegan.”

### S. maltophilia culture conditions.

Strain FLR19 was isolated from the sputum of an adult CF patient in San Diego, CA. Strains CV 2008, FMa 2012, GC 2011, ZC 2005, and ZC 2006 were isolated from the sputum of four adult CF patients in Italy (24). Strains K279a and NCTC 10257 were also included to represent non-CF human isolates. For the growth curves, all eight strains of S. maltophilia were grown in pH 5, 7, and 9 phosphate-buffered Todd-Hewitt broth (THB). The strains were also grown in pH 5 THB spiked with citric acid, lactic acid, or sulfuric acid. In order to determine how pH affects antibiotic resistance, we also grew S. maltophilia cultures under the same conditions but with a concentration gradient of gentamicin (200, 400, or 800 mg/liter), meropenem (64, 128, or 256 mg/liter), and tobramycin (16, 32, or 64 mg/liter) for 24 h. An ANOVA with *post hoc* pairwise comparisons and Bonferroni corrections was conducted in R to compare growth in pH 5 or pH 9 to that in pH 7 medium with and without antibiotics. The fold change in S. maltophilia growth with antibiotics compared to growth without antibiotics was calculated, and a two-sample *t* test with Bonferroni corrections was used to identify significant changes in the fold change values of acidic and basic pH relative to neutral pH. Growth curves were collected using a SpectraMax 190 spectrometer from Molecular Devices. All three antibiotics were purchased from Fisher Scientific. For the transcriptomics and metabolomics experiments, S. maltophilia FLR19 was grown in pH 5, pH 7, or pH 9 phosphate-buffered THB for 24 h. For the FLR19 experiments, culture pH was measured with colorPhast pH strips from EMD Millipore. FLR19 was also grown in phosphate-buffered pH 5, 7, and 9 artificial sputum media (ASM) based on a recipe from Palmer et al. (49, 50). For the ASM growth curves, colony counts of spot dilution plates were used to calculate FLR19 concentration.

### Preparation of S. maltophilia FLR19 transcriptomes and metabolomes.

The cells were centrifuged and pellets were stored in TRIzol at −80˚C for RNA sequencing. RNA was extracted using the Zymo Direct-zol miniprep kit and concentrated with the Zymo RNA Clean and Concentrate kit. An Illumina Ribo-Zero rRNA removal kit was used to remove rRNA. The RNA libraries were prepared for sequencing with the TruSeq RNA sample preparation kit. Paired-end reads (250 bp) were sequenced on an Illumina HiSeq instrument. For metabolomics analysis, the supernatant was stored at −80˚C (51). Triplicates of the uninoculated media and supernatants of acidic, neutral, and basic FLR19 cultures were sent to the West Coast Metabolomics Center for untargeted metabolomics analysis performed with gas chromatography-time of flight mass spectrometry (GC-TOF-MS). Metabolites were extracted from the bacterial supernatants with a 3:3:2 mixture of isopropanol, acetonitrile, and water. The GC-MS analysis followed Fiehn lab standard operating procedures (52).

### S. maltophilia FLR19 metabolomics analysis.

The Bray-Curtis distances of the metabolite intensities for all samples were calculated using the “vegdist” function from the R package vegan and visualized with a principal coordinate analysis (PCoA) plot using the “pcoa” function from the R package “ape.” In order to identify metabolites that were shared among or unique to the S. maltophilia FLR19 acidic, neutral, and basic metabolomes, the average normalized intensity for the metabolites in the three uninoculated replicates was calculated. The uninoculated media averages were subtracted from the metabolite intensities from the acidic, neutral, and basic metabolomes. A metabolite was considered to be produced in a certain condition if the blank-subtracted metabolite intensity was positive for all three replicates and consumed if the blank-subtracted metabolite intensity was negative for all three replicates. Metabolites with significantly different levels in the acidic or basic metabolomes, compared to the neutral metabolome, were identified with a Kruskal-Wallis ANOVA and *post hoc* Dunn comparisons in R. Volcano plots were made in R to depict the fold change in metabolite abundances and *P* values of the *post hoc* Dunn comparisons of the metabolites considered to be significant from the Kruskal-Wallis ANOVA.

### Transcriptomic analysis.

All RNA sequencing preprocessing was performed on the UC Irvine High Performance Computer Cluster in a Linux environment. Reads were quality-filtered using Trimmomatic version 0.35. (53). Specifically, adaptors were trimmed from the ends of reads, and the parameters used for filtering were as follows: minimum read length of 50 bp and a 4-bp sliding window average Phred quality score of 20. Overlapping reads were combined using Paired-End reAd mergeR (PEAR) and processed as single-end reads, separate from the remaining paired-end reads (54). The preprocessed reads were then aligned to the S. maltophilia FLR19 genome using Bowtie 2 in single-end or paired-end mode (55). In order to identify differentially expressed genes in the acidic versus basic transcriptomes (using the neutral transcriptome as a reference), HTSeq-Count (56) and the R package edgeR (57) were used to count the number of reads aligned to a gene. The sum counts of the overlapping reads processed as single-end reads and paired-end reads were calculated for each gene. Genes with log_2_ fold changes greater than 1 or less than −1 and FDR values of <0.05 were considered to be differentially expressed.

### S. maltophilia metatranscriptome and transcriptome comparison.

In order to identify genes that were expressed by S. maltophilia in cystic fibrosis sputum, seven cystic fibrosis metatranscriptomes (27) were quality filtered using Trimmomatic version 0.35 with the following parameters: minimum read length of 35 and a 4-bp sliding window with an average Phred score of 20 (53). The metatranscriptome reads were then dereplicated with Prinseq-lite version 0.20.4 (58). To ensure that stringent alignment parameters were used, the quality filtered metatranscriptome reads were first aligned using Bowtie 2 (55) to a custom-made database consisting of all non-S. maltophilia bacterial genomes associated with cystic fibrosis patients from PATRIC (1,812 genomes) (33). Reads that did not align to the CF bacterial database were then mapped to the S. maltophilia pangenome consisting of all the coding sequences from the 74 strains from our phylogenomics with Bowtie 1.

In order to compare gene expression of the *in vitro* S. maltophilia FLR19 transcriptomes to that of the metatranscriptomes, we processed the *in vitro* reads using a similar pipeline as that for the metatranscriptomes. Briefly, overlapping paired-end reads from the transcriptomes were aligned to the pangenome as a single read. The number of reads that aligned to a gene was then counted in R and the RPKM (reads per kilobase of transcript per million mapped reads) values were calculated. Reads that mapped to multiple genes were included in the quantification, since the same read often mapped to core genes across multiple genomes. The RPKM values from genes expressed in all nine samples were used to build a Euclidean distance matrix with the “vegdist” function from the R package vegan. The distance matrix coordinates were plotted on a principal-coordinate analysis (PCoA) plot. A nested PERMANOVA was used to compare the gene RPKM values using the “adonis” function in vegan. For the PERMANOVA, the design structure nested experimental condition (acidic, basic, or neutral pH or sputum) in the source of the sample (*in vitro* versus *in vivo*). The proportion of CF database-filtered reads that aligned to a functional category was determined by counting the reads that mapped to genes found in the RAST SEED cellular categories (29). The *in vitro* FLR19 transcriptome reads were filtered using the same pipeline to compare the proportion of reads that mapped to the SEED categories *in vitro* to CF sputum.

### Data availability.

The assembled FLR19 genome is publicly available on the PATRIC website under accession number 40324.190 (33). The metabolomics data are available in Data Set S4. The RNA sequencing reads are deposited in the NCBI GEO database under accession number GSE121347, and the analyses can be found in Data Sets S5 to S7. The metatranscriptome reads can be found in the Sequence Read Archive under accession number SRP135669 (27).

## Supplementary Material

Supplemental file 1

Supplemental file 2

Supplemental file 3

Supplemental file 4

Supplemental file 5

Supplemental file 6

Supplemental file 7

Supplemental file 8
