# Supplementary material for: Cystic Fibrosis-Associated Stenotrophomonas maltophilia Strain-Specific Adaptations and Responses to pH
Source: J Bacteriol. 2019 Mar 13;201(7):e00478-18. doi: 10.1128/JB.00478-18 (PMC6416904; doi:10.1128/JB.00478-18)
Supplement: Supplemental file 8 [file JB.00478-18-s0008.pdf]

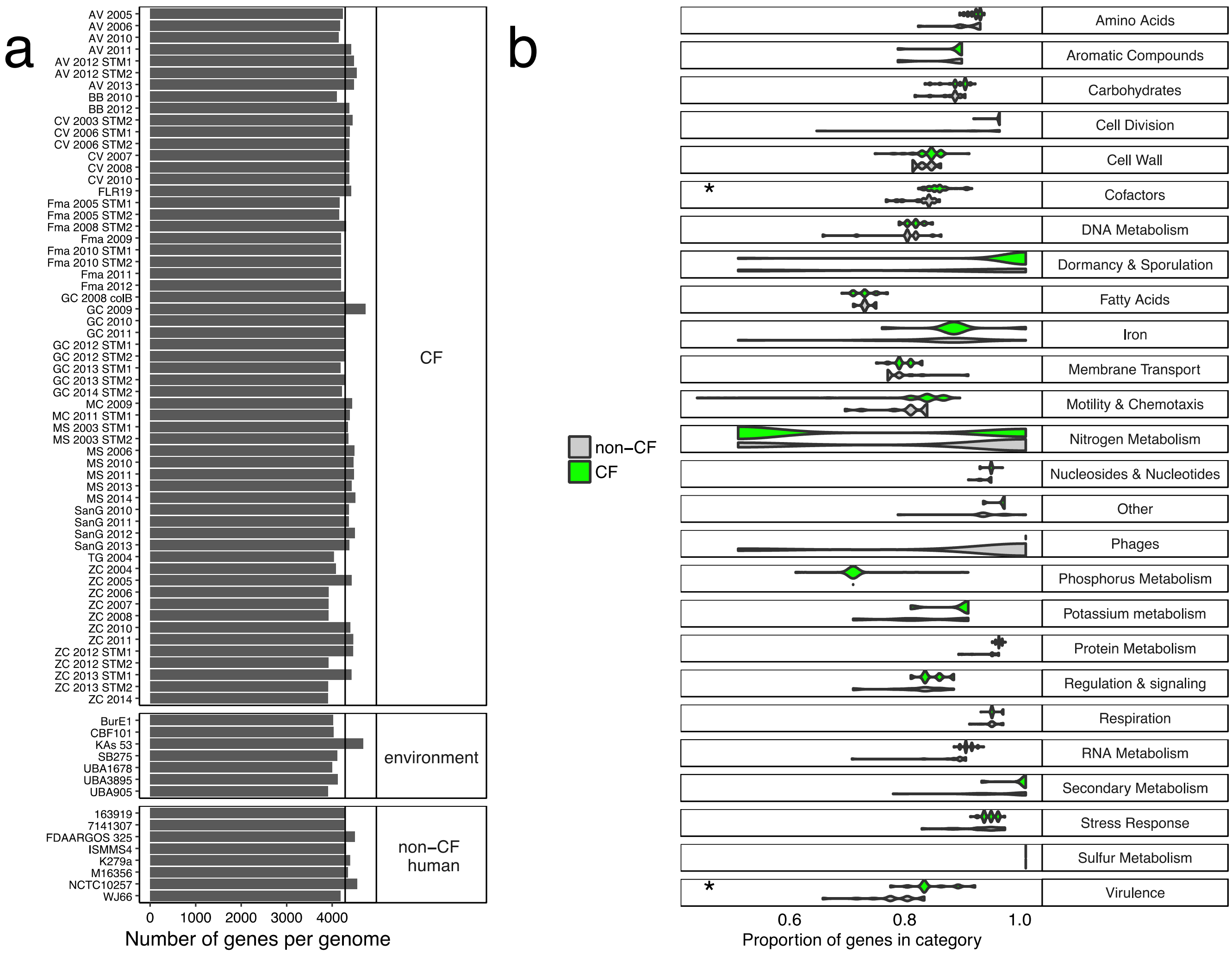

Fig. S1: (a) The number of genes in the 74 *S. maltophilia* strains ranged from 3,908 (strain UBA905) to 4,733 genes (strain GC 2009), with an average of 4,285 genes per genome. (b) Functional potential of *S. maltophilia* CF (N=59) and non-CF (N=15) strains. The mean proportion of genes found in CF and non-CF strains from one of 26 functional categories. Asterisks indicate categories significantly enriched for in the CF strains, t-test with Bonferroni corrections, p-value<0.05.

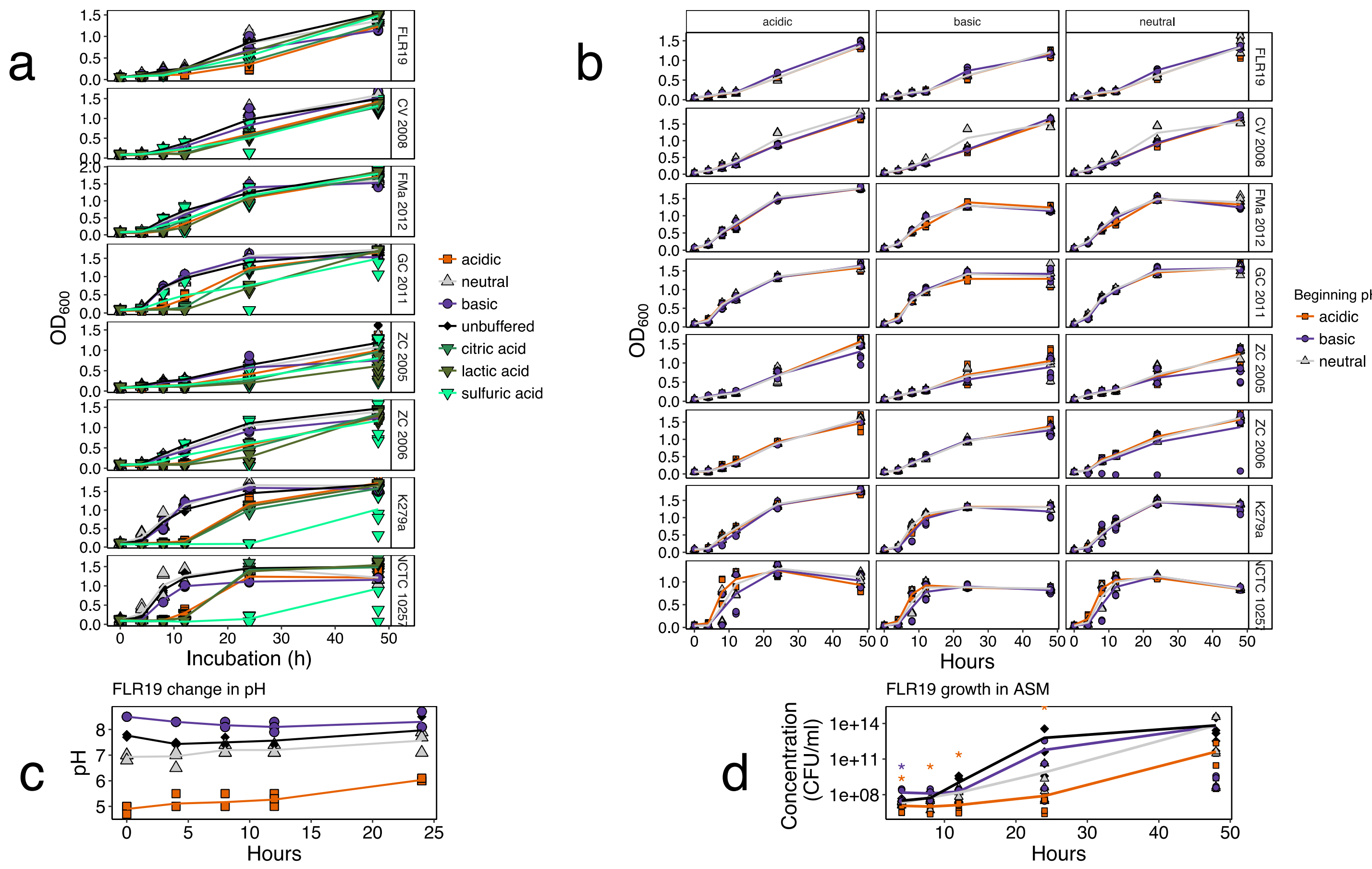

Fig. S2: (a) The growth of eight *S. maltophilia* strains, consisting of six CF (FLR19, CV 2008, FMa 2012, GC 2011, ZC 2005, and ZC 2006) and two non-CF, human isolates (K279a, NCTC 10257) in pH 5 (orange square), pH 7 (gray triangle), or pH 9 (purple circle) phosphate-buffered Todd-Hewitt broth and Todd-Hewitt broth spiked with citric acid, lactic acid, or sulfuric acid (green shades of upside-down triangle) to reach a pH of 5 (N=6-9 replicates). (b) pH cross-over experiments indicate that growth for 24h in one pH does not select for more acidic or basic pH tolerant cells. The colors and shapes represent the initial pH culture conditions (pH 5 = orange square, pH 7 = gray triangle, or pH 9 = purple circle), while the vertical column headers indicate the pH of the crossover experiment (N=6 replicates). (c) *S. maltophilia* FLR19 cultures change in pH over time (N=3-4 replicates). (d) *S. maltophilia* FLR19 growth (CFU/ml) in phosphate buffered artificial sputum media (N=5-6 replicates). By the 24h time point, most of the strains were forming

aggregative biofilms, but we were still able to see distinctions in growth with the dilution spot plating method.

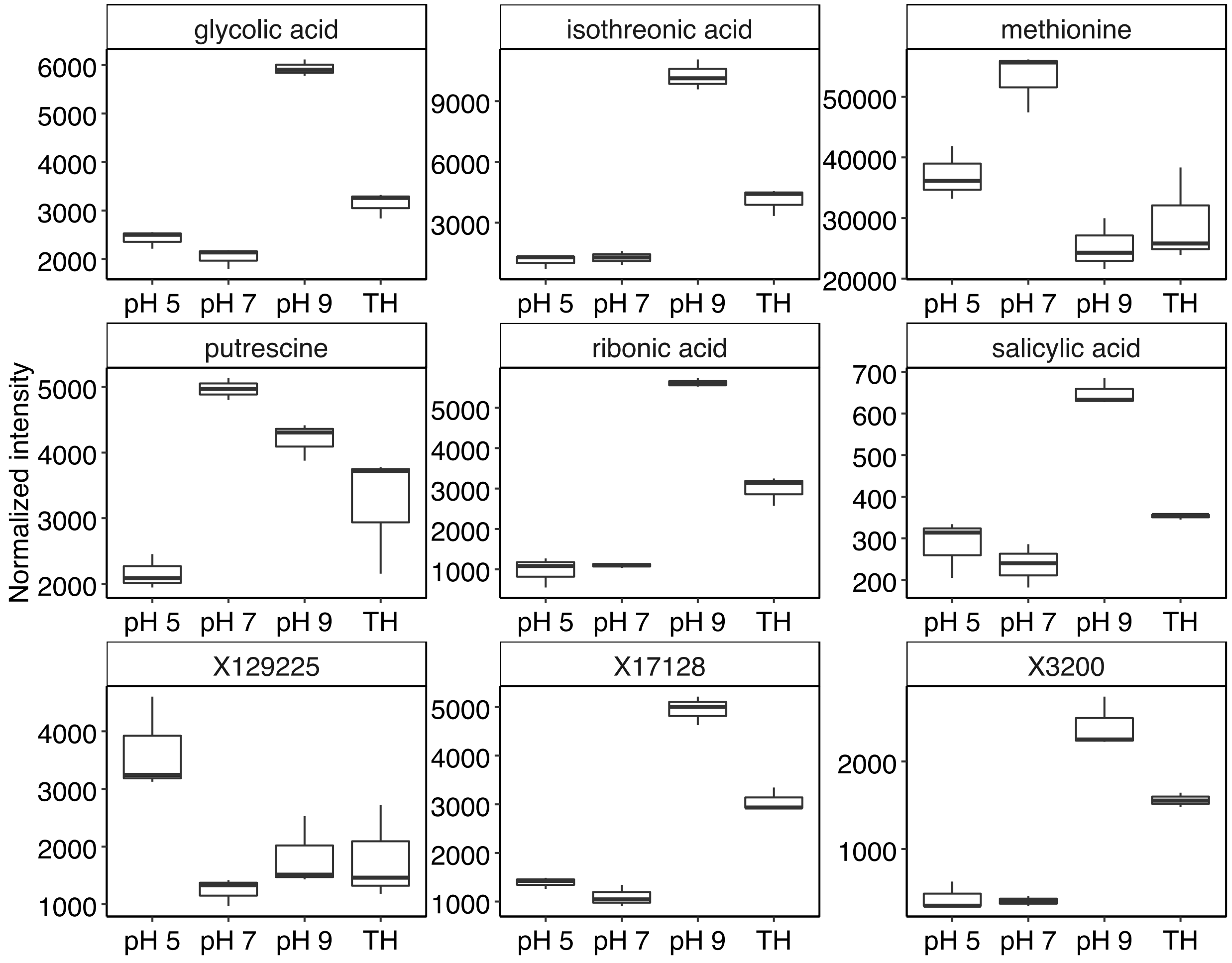

Fig. S3: Abundances of metabolites with significantly higher or lower levels in the acidic metabolome and basic metabolome compared to the neutral metabolome (N = 3 per condition, bars represent the mean abundance).

SUPPLEMENTAL MATERIALS LEGENDS

Dataset S1: Genomes and corresponding meta-data used in phylogenomics analyses (N=153 genomes).

Dataset S2: Presence-absence matrix of gene clusters (N=14,136) at a 95% amino acid identity used in pan-genome analyses (N=74 genomes). The colors of the strain names indicate isolation source, where brown = terrestrial; blue = aquatic; green = CF sputum; and red = non-CF human.

Dataset S3: Gene groupings into cellular categories from the RAST SEED annotations of strain FLR19 (CF) and K279a (non-CF, human) in the blue columns. The middle gray columns show the mean proportion of genes from a cellular category for CF (N=59) and non-CF (N=15) strains from our pan-genome analyses. The proportion of transcriptome reads from the *in vitro* experiments and CF sputum that aligned to RAST SEED cellular categories from strain FLR19 in the green columns.

Dataset S4: Metabolomics data from FLR19 grown in acidic, basic, or neutral pH and uninoculated blank THB (N=3 replicates).

Dataset S5: Number of reads from the transcriptomics and metatranscriptomics pipeline.

Dataset S6: Differentially expressed genes from *in vitro* FLR19 pH experiment.

Dataset S7: RPKM values of transcriptome and metatranscriptome reads that aligned to the *S. maltophilia* pan-genome (N=14,136 genes).
